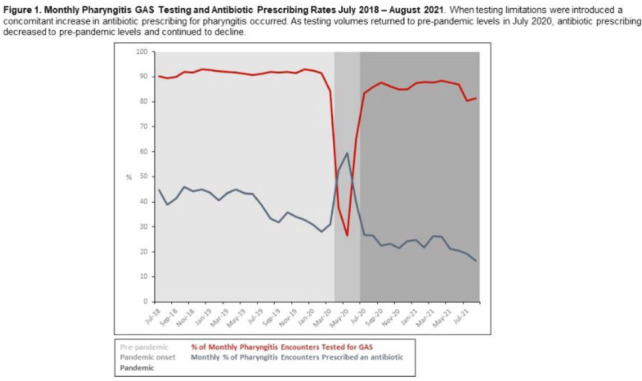# Rapid streptococcal pharyngitis testing and antibiotic prescribing before and during the COVID-19 pandemic

**DOI:** 10.1017/ash.2022.91

**Published:** 2022-05-16

**Authors:** Allan Seibert, Eddie Stenehjem, Anthony Wallin, Park Willis, Kim Brunisholz, Naresh Kumar, Valoree Stanfield, Nora Fino, Daniel Shapiro, Adam Hersh

## Abstract

**Background:** Pharyngitis is 1 of the most common conditions leading to inappropriate antibiotic prescriptions. When personal protective equipment (PPE) was at first constrained during the COVID-19 pandemic, Intermountain Healthcare recommended limiting rapid group A streptococcal pharyngitis (GAS) testing in urgent-care clinics to preserve PPE. Notably, the percentage of pharyngitis encounters prescribed an antibiotic and that underwent GAS testing is a key Healthcare Effectiveness Data and Information Set (HEDIS) measure. We have described our experience with urgent-care pharyngitis encounters and the impact of temporarily reducing GAS testing on antibiotic prescribing before and during the COVID19 pandemic. **Method:** We identified all urgent care encounters between July 2018 and August 2021 associated with a primary diagnosis of pharyngitis using ICD-10 CM codes and a validated methodology. Pharyngitis encounters were assessed for antibiotic prescriptions ordered through the electronic health record (EHR) and the use of point-of-care rapid GAS tests. Pharyngitis encounters were analyzed monthly. We assessed the percentage of encounters associated with an antibiotic prescription regardless of testing and the percentage of encounters associated with an antibiotic prescription when a GAS test was or was not performed. We examined 3 periods relating to COVID-19 and GAS testing recommendations: the prepandemic period (July 2018–March 2020), the pandemic onset period (April 2020–June 2020), and the pandemic period (July 2020–August 2021). **Results:** Prior to the pandemic, the monthly percentage of pharyngitis encounters for which rapid GAS testing was performed was nearly 90% (Fig. 1). The average monthly percentage of urgent-care pharyngitis encounters prescribed an antibiotic was 38.9%, and the average percentage of monthly pharyngitis encounters prescribed an antibiotic that also underwent GAS testing was 90.4%. This HEDIS measure declined from 90.4% during the prepandemic period to 29.8% in the pandemic onset period when GAS testing was limited. Following resumption of routine testing practices the monthly percentage of urgent-care pharyngitis encounters for which rapid GAS testing was performed returned to levels ≥80% by July 2020 (Fig. 1). The average percentage of monthly pharyngitis encounters prescribed an antibiotic that also underwent GAS testing rose to 87.3% during this period. **Conclusions:** Limited PPE in our urgent care centers during the initial months of the COVID-19 pandemic was associated with a mandated substantial decline in rapid GAS testing. As testing volume decreased, we noted a simultaneous relative increase of >30% in antibiotic prescribing for pharyngitis. These findings suggest that rapid streptococcal testing promotes appropriate antibiotic prescribing.

**Funding:** None

**Disclosures:** None

**Figure:**